# What matters and to whom: the politics of scientific impact

**DOI:** 10.1590/1518-8345.0000.5123

**Published:** 2026-10-09

**Authors:** Baptiste Godrie

**Affiliations:** 1 Université de Sherbrooke, Sherbrooke, Quebec, Canada. baptiste.godrie@usherbrooke.ca

**Figure:**
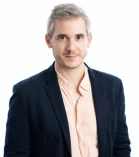

Researchers are increasingly being asked by research funding agencies to demonstrate the impact of their work using seemingly objective indicators. These indicators allow research funding agencies to justify the use of public funds and enable universities to demonstrate their influence. It is also a matter of making the contributions of research to public policy, professional practice, and collective well-being across various sectors of society more visible and demonstrable.

The indicators commonly used to assess these impacts vary depending on the context and how impact is defined. Some, such as the number of scientific citations, journal impact factors, and the amount of funding secured, focus on the scientific recognition of research. Others aim to document its influence beyond the academic world. In the field of health, these include indicators such as the incorporation of findings into clinical guidelines, the number of institutions that have adopted an organizational innovation, and changes to care and treatment protocols, the number of trained professionals, and the measurable improvements in health outcomes.

However, the seemingly objective nature of these indicators tends to obscure the value judgments underlying their selection: which changes deserve to be counted, over what time frame, and from the perspective of which stakeholders? Which outcomes are highlighted by these indicators, and which are overlooked? My perspective on these issues is rooted in a range of my academic experiences that have led me to recognize that the evaluation of the impact of scientific work involves deeply political choices about which forms of knowledge, social actors, and transformations are deemed worthy of recognition. First, it stems from my background as a sociologist specializing in social inequalities, conducting research with professionals and marginalized communities in the fields of mental health and poverty in Quebec province (Canada). This experience has led me to reflect on the importance of recognizing not only the scientific contributions of research but also its ethical and political contributions. Second, it is informed by my experience as scientific director of two research infrastructures funded by the *Fonds de recherche du Québec* and the Quebec Ministry of Health and Social Services. The coexistence of these funding sources, each with distinct expectations - one emphasizing contributions to the scientific field and the other to the development of effective and innovative practices - led me to reflect on how research impact can be highlighted and recognized both within and beyond academic institutions. The reflections that follow are grounded in these experiences.

First and foremost, thinking about the impact of research requires moving away from indicators focused on individual and short-term impacts. Some transformations are slow, long-term, and collective. Much research in the humanities and social sciences on racism, gender, and social class contributes to the deconstruction of persistent neoliberal explanations that attribute full responsibility for success or failure to individuals or groups, while obscuring the social relations that help produce these outcomes. Feminist perspective on the world, and particularly on health and care, which has been shaped over the long term, provide a powerful example here: it has replaced individualistic narratives with an analysis of gendered social relations and practices of resistance in the face of domination. Reframing “crimes of passion” as femicides, “flirting” as sexual harassment, and “devotion” and “sacrifice” as care work and emotional labor constitutes a crucial transformation of the world that impact indicators are unable to capture. Such shifts from dominant understanding to previously (and often still) marginalized interpretations of the world cannot be attributed solely to scientific research, even though they contribute significantly to it, which makes it difficult - if not impossible - to attribute its origin to a specific person or group of people and their work.

Furthermore, participatory research highlights the need to move away from the institutional academic criteria often used to identify research impacts. Such research resists the broader trend toward academic neoliberalism to prioritize, above all else in academic careers, forms of impact that can be published and peer-reviewed, reducing research to the production of supposedly “objective” data and results. Several studies on participatory research identify, in addition to scientific criteria for the quality of the knowledge produced, ethical and political criteria that help bring to light impacts that are usually given little, if any, consideration. In particular, this involves determining the extent to which participatory research contributes to the redistribution of financial and material resources among participants, to the recognition of their intellectual contribution to the knowledge produced, and to improving people’s lived experiences and increasing their capacity for action.

Nursing, as both a discipline and practice, offers fertile ground for reflecting on these issues, particularly in Latin America, where traditions of collective health (*salud colectiva*/*saúde coletiva*) have a significant influence on the field’s development. Its contribution to care, to collaborative knowledge production with communities, and to improving living conditions often yields benefits that go beyond conventional performance indicators. Several Latin American nursing traditions view care as both a clinical intervention and a practice of justice, combining awareness-raising and social transformation.

The fact that such impacts go unnoticed in academic careers is by no means accidental: it is the result of scientific institutions and policies that prioritize short-term timeframes, which tend to overlook slow, relational, and collective transformations by relying on standardized quantitative indicators that exclude impacts not attributable to a funded project. Rooted in a system that seeks to align research with the imperatives of productivity and competition, this invisibility can undermine communities that do not receive the institutional recognition they deserve, while also hindering collective learning. Furthermore, not everything can be measured, and many impacts will remain unknown, including avoided negative impacts, such as preventing a decline in health, reducing stigma, or preventing the adoption of harmful public policies.

Several frameworks, including Research Quality Plus^1^, call for shifting the focus from solely measurable impacts to criteria such as the social relevance of research, the inclusion of affected populations and the recognition of their knowledge, the contribution of research to collective transformations, and the conditions that enable the social appropriation of the knowledge produced.

The problem, therefore, is not only that certain impacts go unmeasured, but also that the tools used to document them reflect a particular conception of what deserves to be recognized as a significant scientific and social contribution. Documenting research impacts therefore presents significant challenges that scholars must address in dialogue with communities and research funding agencies, who have a responsibility to adapt their practices to better reflect the complexity of the social transformations that research fosters or supports.
