# Supplementary material for: What matters and to whom: the politics of scientific impact
Source: Rev Lat Am Enfermagem. 2026 Oct 9;34:e5123. doi: 10.1590/1518-8345.0000.5123 (PMC13652533; doi:10.1590/1518-8345.0000.5123)
Supplement: Translation

# Translation: O que importa e para quem: a política do impacto científico

**DOI:** 10.1590/1518-8345.0000.5124

**Authors:** Baptiste Godrie

**Affiliations:** 1 Université de Sherbrooke, Sherbrooke, Quebec, Canadá. baptiste.godrie@usherbrooke.ca

Pesquisadores têm sido cada vez mais solicitados por agências de fomento à pesquisa a demonstrar o impacto de seus trabalhos utilizando indicadores aparentemente objetivos. Esses indicadores permitem que as agências de fomento justifiquem o uso de recursos públicos e possibilitam às universidades demonstrar sua influência. Trata-se, também, de tornar mais visíveis e demonstráveis as contribuições da pesquisa para as políticas públicas, a prática profissional e o bem-estar coletivo em diversos setores da sociedade.

Os indicadores comumente utilizados para avaliar esses impactos variam conforme o contexto e a forma como o impacto é definido. Alguns, como o número de citações científicas, os fatores de impacto dos periódicos e a quantidade de financiamento obtida, focam no reconhecimento científico da pesquisa. Outros visam documentar sua influência além do mundo acadêmico. Na área da saúde, estes incluem indicadores como a incorporação de achados em diretrizes clínicas, o número de instituições que adotaram uma inovação organizacional, as mudanças nos protocolos de cuidado e tratamento, o número de profissionais capacitados e as melhorias mensuráveis nos resultados de saúde.

No entanto, a natureza aparentemente objetiva desses indicadores tende a ocultar os julgamentos de valor subjacentes à sua seleção: quais mudanças merecem ser contabilizadas, em qual horizonte temporal e sob a perspectiva de quais partes interessadas (*stakeholders*)? Quais resultados são destacados por esses indicadores e quais são negligenciados? Minha perspectiva sobre essas questões está fundamentada em um conjunto de experiências acadêmicas que me levaram a reconhecer que a avaliação do impacto do trabalho científico envolve escolhas profundamente políticas sobre quais formas de conhecimento, atores sociais e transformações são considerados dignos de reconhecimento. Primeiro, decorre da minha trajetória como sociólogo especializado em desigualdades sociais, realizando pesquisas com profissionais e comunidades marginalizadas nos campos da saúde mental e da pobreza na província de Quebec (Canadá). Essa experiência me levou a refletir sobre a importância de reconhecer não apenas as contribuições científicas da pesquisa, mas também suas contribuições éticas e políticas. Segundo, é informada pela minha experiência como diretor científico de duas infraestruturas de pesquisa financiadas pelo *Fonds de recherche du Québec* e pelo Ministério da Saúde e Serviços Sociais de Quebec. A coexistência dessas fontes de financiamento, cada uma com expectativas distintas - uma enfatizando as contribuições para o campo científico e a outra para o desenvolvimento de práticas eficazes e inovadoras -, levou-me a refletir sobre como o impacto da pesquisa pode ser destacado e reconhecido tanto dentro quanto fora das instituições acadêmicas. As reflexões que se seguem estão ancoradas nessas experiências.

Em primeiro lugar, pensar sobre o impacto da pesquisa exige afastar-se de indicadores focados em impactos individuais e de curto prazo. Algumas transformações são lentas, de longo prazo e coletivas. Grande parte das pesquisas nas ciências humanas e sociais sobre racismo, gênero e classe social contribui para a desconstrução de explicações neoliberais persistentes que atribuem total responsabilidade pelo sucesso ou fracasso aos indivíduos ou grupos, enquanto ocultam as relações sociais que ajudam a produzir esses resultados. A perspectiva feminista sobre o mundo, e particularmente sobre a saúde e o cuidado, moldada ao longo do tempo, fornece um exemplo emblemático a esse respeito: ela substituiu narrativas individualistas por uma análise das relações sociais de gênero e das práticas de resistência diante da dominação. Reenquadrar “crimes de paixão” como feminicídios, “paquera” como assédio sexual, e “dedicação” e “sacrifício” como trabalho de cuidado (*care work*) e trabalho emocional constitui uma transformação crucial do mundo que os indicadores de impacto são incapazes de capturar. Tais mudanças, que vão de interpretações dominantes para interpretações do mundo anteriormente (e muitas vezes ainda) marginalizadas, não podem ser atribuídas exclusivamente à pesquisa científica, muito embora esta contribua significativamente para elas. Isso torna difícil - se não impossível - atribuir sua origem a uma pessoa ou grupo específico de pessoas e aos seus trabalhos.

Além disso, a pesquisa participativa evidencia a necessidade de se afastar dos critérios acadêmicos institucionais frequentemente utilizados para identificar os impactos da pesquisa. Esse tipo de pesquisa resiste à tendência mais ampla do neoliberalismo acadêmico de priorizar, acima de qualquer outra coisa nas carreiras acadêmicas, formas de impacto que possam ser publicadas e revisadas por pares, reduzindo a pesquisa à produção de dados e resultados supostamente “objetivos”. Diversos estudos sobre pesquisa participativa identificam, além dos critérios científicos para a qualidade do conhecimento produzido, critérios éticos e políticos que ajudam a trazer à luz impactos que costumam receber pouca ou nenhuma consideração. Em particular, isso envolve determinar em que medida a pesquisa participativa contribui para a redistribuição de recursos financeiros e materiais entre os participantes, para o reconhecimento de sua contribuição intelectual ao conhecimento produzido e para a melhoria das experiências vividas pelas pessoas e o aumento de sua capacidade de ação.

A Enfermagem, como disciplina e prática, oferece um terreno fértil para refletir sobre essas questões, especialmente na América Latina, onde as tradições da Saúde Coletiva exercem uma influência significativa no desenvolvimento do campo. Sua contribuição para o cuidado, para a produção colaborativa de conhecimento com as comunidades e para a melhoria das condições de vida frequentemente gera benefícios que vão além dos indicadores convencionais de desempenho. Diversas tradições da enfermagem latino-americana concebem o cuidado tanto como uma intervenção clínica quanto como uma prática de justiça, articulando conscientização e transformação social.

O fato de tais impactos passarem despercebidos nas carreiras acadêmicas não é, de forma alguma, acidental: é o resultado de políticas e instituições científicas que priorizam horizontes temporais de curto prazo, as quais tendem a ignorar transformações lentas, relacionais e coletivas ao se apoiarem em indicadores quantitativos padronizados que excluem impactos não atribuíveis a um projeto financiado específico. Enraizada em um sistema que busca alinhar a pesquisa aos imperativos da produtividade e da competição, essa invisibilidade pode prejudicar comunidades que não recebem o reconhecimento institucional que merecem, ao mesmo tempo em que compromete a aprendizagem coletiva. Além disso, nem tudo pode ser medido, e muitos impactos permanecerão desconhecidos, incluindo os impactos negativos evitados - como prevenir o declínio da saúde, reduzir o estigma ou evitar a adoção de políticas públicas prejudiciais.

Vários arcabouços, incluindo o *Research Quality Plus* ^1^, defendem o deslocamento do foco de impactos puramente mensuráveis para critérios como a relevância social da pesquisa, a inclusão das populações afetadas e o reconhecimento de seus conhecimentos, a contribuição da pesquisa para transformações coletivas e as condições que possibilitam a apropriação social do conhecimento produzido.

O problema, portanto, não é apenas que certos impactos deixem de ser medidos, mas também que as ferramentas utilizadas para documentá-los refletem uma concepção particular do que merece ser reconhecido como uma contribuição científica e social significativa. Documentar os impactos da pesquisa apresenta, assim, desafios expressivos que os pesquisadores devem enfrentar em diálogo com as comunidades e as agências de fomento à pesquisa - estas últimas com a responsabilidade de adaptar suas práticas para refletir melhor a complexidade das transformações sociais que a pesquisa estimula ou sustenta.

# Translation: Qué cuenta y para quién: la política del impacto científico

**DOI:** 10.1590/1518-8345.0000.5122

**Authors:** Baptiste Godrie

**Affiliations:** 1 Université de Sherbrooke, Sherbrooke, Quebec, Canadá. baptiste.godrie@usherbrooke.ca

Los investigadores suelen recibir la solicitud de los organismos financiadores de la investigación de identificar los impactos de sus trabajos mediante indicadores aparentemente objetivos. Estos permiten a los organismos financiadores justificar el uso de los fondos públicos y a las universidades demostrar su impacto. También se trata de hacer más visibles y demostrables ante los distintos sectores de la sociedad las contribuciones de la investigación al desarrollo de las políticas públicas, las prácticas profesionales y el bienestar colectivo.

Los indicadores que se utilizan habitualmente para evaluar estos efectos varían según los contextos y las concepciones del impacto. Algunos, como el número de citas científicas, el factor de impacto de las revistas y los montos de financiamiento obtenidos, se centran en el reconocimiento científico de los trabajos. Otros proponen documentar su influencia más allá del ámbito académico. En el ámbito de la salud, se trata de indicadores como el número de establecimientos que han adoptado una innovación organizacional, la modificación de los protocolos de atención, el número de profesionales capacitados, así como la reducción de ciertos indicadores de salud.

Sin embargo, el carácter aparentemente objetivo de estos indicadores tiende a ocultar las decisiones de valor que rigen su selección: ¿qué cambios merecen ser contabilizados, en qué horizonte temporal y desde la perspectiva de qué actores? ¿Cuáles son los impactos que estos indicadores ponen de relieve y cuáles son los que se dejan de lado?

Mi punto de vista sobre estos temas se basa en experiencias académicas que me llevan a reconocer que la evaluación de los impactos de los trabajos científicos implica decisiones profundamente políticas respecto a las formas de conocimiento, los actores y las transformaciones que se consideran dignas de reconocimiento. Se trata, en primer lugar, de mi trayectoria como sociólogo especializado en desigualdades sociales, durante la cual he realizado investigaciones con profesionales y comunidades marginadas en el ámbito de la salud mental y la pobreza en Quebec (Canadá). Esta experiencia inspira mis reflexiones sobre la importancia de reconocer, además de los impactos científicos, los impactos éticos y políticos de la investigación. En segundo lugar, se trata de mi experiencia como director científico de dos infraestructuras de investigación financiadas por los *Fonds de recherche du Québec* y el Ministerio de Salud y Servicios Sociales. La coexistencia de estos dos financiadores con expectativas distintas; uno que valora las contribuciones al conocimiento científico y otro que prioriza el desarrollo de prácticas eficaces e innovadoras; me llevó a reflexionar sobre las formas de visibilizar los impactos de la investigación tanto dentro como fuera de las instituciones universitarias. A partir de estas experiencias, propongo las siguientes reflexiones.

En primer lugar, reflexionar sobre los impactos de la investigación exige romper con los indicadores centrados en los impactos individuales y a corto plazo. Algunas transformaciones son lentas, a largo plazo y colectivas. Numerosos estudios de las ciencias humanas y sociales sobre el racismo, el género y las clases sociales contribuyen a cuestionar las persistentes explicaciones neoliberales que consisten en atribuir a los individuos o a los grupos toda la responsabilidad de su éxito o fracaso, al tiempo que naturalizan las relaciones sociales que contribuyen a generarlos. La lectura feminista del mundo y, en particular, de la salud y del *care* (cuidados), forjada a lo largo del tiempo, resulta aquí elocuente: ha sustituido los relatos individualistas por un análisis de las relaciones sociales de género y de las prácticas de resistencia frente a las dominaciones. La reclasificación de los “crímenes pasionales” como feminicidios, del “coqueteo” como acoso sexual y de la “dedicación” y el “sacrificio” como *care* y trabajo emocional constituye una transformación crucial del mundo que los indicadores de impacto no logran captar. Esas transformaciones que desplazan las concepciones dominantes hacia nuevas formas de interpretar el mundo no se pueden atribuir únicamente a los trabajos científicos, aunque estos contribuyan de manera significativa a ellas, lo que plantea la dificultad, o incluso la imposibilidad, de atribuir su origen a una persona o a un grupo de personas y a sus trabajos.

Luego, las investigaciones participativas ponen de manifiesto la necesidad de alejarse de los criterios académicos institucionales que suelen utilizarse para identificar los impactos. Estas investigaciones constituyen una forma de resistencia frente a la tendencia del neoliberalismo académico de valorar, ante todo, para el desarrollo de las carreras académicas, únicamente los impactos que pueden publicarse y ser evaluados por pares, y de reducir la investigación a la producción de datos y resultados “objetivos”.

Varios estudios sobre la investigación participativa identifican, además de los criterios científicos de calidad del conocimiento generado, criterios éticos y políticos que permiten visibilizar repercusiones que, por lo general, se tienen poco en cuenta o que ni siquiera se consideran. Se trata, en particular, de identificar en qué medida las investigaciones realizadas contribuyen a una redistribución de los recursos financieros y materiales entre los participantes, al reconocimiento de su contribución intelectual a los conocimientos generados, así como a la mejora de las condiciones de vida de las personas y al aumento de su capacidad de acción.

Las ciencias de la enfermería ofrecen un terreno fértil para reflexionar sobre estos temas, especialmente en América Latina, donde las tradiciones de salud colectiva ejercen una influencia importante en su desarrollo. Su contribución al *care*, a la generación de conocimientos junto con las comunidades y a la mejora de las condiciones de vida suele generar beneficios que van más allá de los indicadores convencionales de desempeño. Así lo demuestran varias tradiciones de enfermería latinoamericanas, que conciben la atención como una intervención clínica y, al mismo tiempo, como una práctica de justicia, que combina la concientización y la transformación social.

La invisibilidad de tales repercusiones en las carreras académicas no es en absoluto casual: está orquestada por instituciones y políticas científicas que dan prioridad a los plazos cortos, lo que tiende a ignorar las transformaciones lentas, relacionales y colectivas mediante el uso de indicadores cuantitativos estandarizados que excluyen las repercusiones que no son atribuibles a un proyecto subvencionado. Esta invisibilidad de los beneficios, generada por un sistema que tiende a disciplinar la investigación para hacerla compatible con las lógicas de productividad y competencia, puede debilitar a las comunidades que no reciben el reconocimiento institucional que merecen y, al mismo tiempo, obstaculizar sus procesos de aprendizaje colectivo. Además, no todo se puede medir, y muchos efectos seguirán siendo desconocidos, incluidos los efectos negativos que se han evitado, como prevenir el deterioro de la salud, reducir la estigmatización o impedir la adopción de una política pública perjudicial.

Varios marcos, entre ellos el *Research Quality Plus* ^1^, proponen, en este sentido, desplazar la atención de los resultados medibles hacia criterios como la relevancia social de la investigación, la inclusión de las poblaciones afectadas y la consideración de sus conocimientos, la contribución de la investigación a las transformaciones colectivas, así como las condiciones que permiten la apropiación social de los conocimientos generados.

El problema no es solo que algunos impactos no se puedan medir, sino también que los instrumentos utilizados para documentarlos reflejan una concepción particular de lo que merece ser reconocido como una contribución científica y social significativa. Por lo tanto, documentar los impactos plantea desafíos que los académicos deben abordar en diálogo con las comunidades y los financiadores de la investigación, quienes tienen la responsabilidad de adaptar sus prácticas para reconocer mejor la complejidad de las transformaciones sociales impulsadas o acompañadas por las investigaciones.
